# Editorial: Tissue and organ decellularization strategies in regenerative medicine; recent advances, current translational challenges, and future directions

**DOI:** 10.3389/fbioe.2023.1201041

**Published:** 2023-04-12

**Authors:** Kamal Hussein, Sotirios Korossis, Laura Iop

**Affiliations:** ^1^ Department of Surgery, Anaesthesiology and Radiology, College of Veterinary Medicine, Assiut University, Assiut, Egypt; ^2^ Cardiopulmonary Regenerative Engineering (CARE) Group, Centre for Biological Engineering (CBE), Wolfson School of Mechanical, Electrical and Manufacturing Engineering, Loughborough University, Loughborough, United Kingdom; ^3^ Department of Cardiac Thoracic Vascular Sciences and Public Health, University of Padua, Padua, Italy

**Keywords:** decellularization, scaffold, recellularization, extracellular matrix, reendothelialization, tissue engineering, regenerative medicine

Several different scaffolds of either synthetic or biological origin have been utilized in regenerative medicine for the engineering of tissues and organs. The ideal scaffold should be biocompatible, non-immunogenic, and easily fabricated. In addition, it should provide mechanical stability, mimicking the role of the natural ECM, whilst providing appropriate microstructure for cellular growth. A variety of different methods have been used over the past few years to fabricate synthetic scaffolds, including electrospinning (Amoroso et al., 2012), knitting (Moreira et al., 2014), and 3D printing (Mosadegh et al., 2015), with a view to creating porous structures that would facilitate cellular infiltration and colonization. Ideally, the scaffold would be degraded by the colonizing cells, which will synthesize their own ECM.

The most commonly used polymers in scaffold fabrication are polylactic acid and polyglycolic acid. However, a variety of different polymers have been used in the field over the years. The main reported advantage of polymeric scaffolds was that they can be fabricated in a well-controlled and reproducible manner that reflects their consistency in terms of mechanical properties, degradation rates, pore size, and surface topographies (McClelland et al., 2012). On the other hand, identifying the correct balance between degradation rates and ECM synthesis, as well as imitating the complex histoarchitectural and biomechanical properties of the native ECM, remain significant challenges in synthetic scaffold fabrication.

Biological scaffolds have been fabricated using reconstituted proteins or other structural components of the native ECM. Such scaffolds have been produced using fibrin (Kaminski et al., 2011; Syedain et al., 2015), collagen (Shi and Vesely, 2003), and hyaluronan (Masters et al., 2005). These biopolymers have been prepared as gels in which cells were suspended (Moreira et al., 2014) prior to forming the scaffold geometry. Although such biological scaffolds have an advantage over synthetic ones due to the natural origin of their constitutive components, source-related immunogenicity, inadequate mechanical properties, and absence of natural histoarchitecture remain grossly unsolved issues.

Decellularized tissue represents the current state-of-the-art in scaffold fabrication, featuring natural histoarchitecture and mechanical integrity, similar to the native tissue. Nevertheless, source-related immunogenicity remains an important hurdle for the wide adoption of decellularized tissue scaffolds in the clinical setting, especially in the case of xenogeneic tissue scaffolds. Decellularization is aimed at removing the cellular and nuclear components, and antigens from the tissue, while minimizing the alterations in the composition, histoarchitecture, biological activity, and mechanical integrity of the remaining ECM (Gilbert et al., 2006). Xenogeneic or allogeneic cellular antigens are recognized as foreign by the host and, therefore, trigger an inflammatory response, which can result in immune-mediated graft rejection. Moreover, although the ECM components are well conserved among species and tolerated by the immune system of the host, even when xenogeneic tissue is used (Gilbert et al., 2006), antigens that can trigger an inflammatory response and immune-mediated graft rejection are not only localized in the cells, but have also been reported to reside within the ECM (Galili, 2001; Galili, 2015). Therefore, decellularization alone might not be sufficient for rendering the tissue completely non-immunogenic. On the other hand, harsh treatments for removing antigens from within the ECM can damage the integrity of the ECM proteins.

In the saga of finding efficacious therapies for the treatment of cardiovascular disease, tissue engineering and/or tissue-guided regeneration approaches based on decellularized scaffolds have the potential to provide excellent clinical alternatives to non-viable devices, especially in the field of heart valve replacement. Nevertheless, there are a number of hurdles that still remain in for the wide clinical translation of decellularized scaffolds in cardiovascular surgery.

The decellularization strategies so far developed for cardiac and skeletal muscles were reviewed by Tan et al., pointing out the key requisites for an effective regenerative medicine therapy to tackle cardiovascular disease and musculoskeletal pathologies. The identification of the right balance between effective cell removal and retention of the original biochemical and biomechanical properties of the native tissue remains a challenging task for many tissues and organs. Focusing on ventricular myocardium, Krishnan et al. discussed the components, methods, and obstacles for its biofabrication, describing the wide variety of cell types that have been proposed for a personalized regenerative medicine approach. Aubin et al. reported on an approach that implanted a decellularized myocardial sleeve onto the surface of an infarcted rat heart model. As early as 4 weeks, the sleeve activated a pro-regenerative genetic program, culminating in scaffold repopulation and integration and, ultimately, improvement in overall cardiac function.

The generation of a competent vascular substitute is one of the challenges in cardiovascular bioengineering. Wang et al. reported that the ideal blood vessel replacement should exhibit several characteristics, such as biodegradability, biocompatibility, ability to integrate and bear typical physiologic conditions, while being able to adapt to somatic growth, and can be manufactured at reasonable cost. Bioengineered large blood vessels are a clinical reality, with mainly bovine-derived decellularized arteries and veins. Oropeza et al. reported on a bioengineered ascending aorta, obtained by bioprinting with a viscous ink that was generated by lyophilized and powdered decellularized hearts and human aortic smooth muscle cells. This bioprinted scaffold was reported to possess biomechanical properties similar to those of an immature, growing aorta and, therefore, has the potential to be used as a pediatric aortic model *in vitro*.

The generation of a small-caliber vascular substitute remains a challenging problem, which has been addressed in this Research Topic. Maintenance of patency *in vivo* is the main issue for arteries with a caliber smaller than 6 mm. In order to overcome this issue, Liu et al. proposed the use of crosslinking with photooxidation and penta-galloyl glucose with a view to increasing the biomechanical and biochemical stability of the decellularized internal mammary artery. The treated acellular bovine-origin was grafted in rabbit models of subcutaneous implantation and abdominal aorta transposition, and demonstrated a 4-month overall patency, no stenosis or calcification, null immune response, good biocompatibility, and cellular repopulation. Surgical intervention for small-caliber arterial substitution is frequently urgent, as in the case of coronary bypass after an ischemic heart attack. Most of the current protocols for the preparation of decellularized tubular scaffolds are generally time-consuming and unable to readily address the immediate clinical demand. In order to bridge this temporal gap, Massaro et al. developed a 5-h decellularization protocol for porcine carotid arteries. *In vitro* tests revealed biomechanical integrity and biocompatibility with rat endothelial cells and human umbilical vein endothelial cells (HUVECs), provided that efficient removal of the decellularization detergent (sodium dodecyl sulfate; SDS) was reached. Sterilization and preservation methodologies for small-caliber arterial grafts still need to be optimized, since decellularization protocols might introduce alterations in the treated tissues. Zia et al. investigated the effects of peracetic acid (PAA) and gamma irradiation and reported depletion of collagen IV and overall deterioration of the decellularized tibial artery scaffold using PAA, while both methods reduced the glycosaminoglycan content. This study demonstrated for the first time the feasibility of combining the decellularized scaffold with an electrospun PCL/PEG sleeve that endowed antimicrobial properties and mechanical stability to the graft.

As mentioned above, decellularized heart valves have been among the first scaffolds to reach the clinical setting. Many protocols have been developed to manufacture decellularized heart valve replacements, but yet, decellularization efficiency has been evaluated following not univocal parameters. Naso and Gandaglia emphasized the need to standardize decellularization procedures and recommended a checklist for quality control of heart valve replacements generated with these treatments. Singh et al. proposed a novel application for decellularization treatments. Aldehyde-treated human hearts that are preserved in anatomopathological tissue banks, have been shown to stiffen and might be less informative in medical education and training. Decellularization could restore the original native biomechanical properties of heart valves for high fidelity *in vitro* simulation.

The generation of whole functional solid organs, including liver, kidney, lungs, or heart, which have an inherent sophisticated structure, still faces a number of challenges that need to be addressed (Hussein et al., 2020). Vascular reconstruction or reendothelialization is the main obstacle to avoiding coagulation and guaranteeing continuous blood flow through the transplanted organ (Hussein et al., 2016). Li et al. reviewed the different techniques that have been developed to enhance the reendothelialization efficiency, promote cell seeding of parenchymal cells, and improve graft functionality during both *in vitro* and *in vivo* perfusion of the bioengineered liver. Fathi et al. investigated an alternative way to overcome the reendothelialization problem through an axial pre-vascularization approach of the decellularized liver and evaluated its suitability for pancreatic islet transplantation. They reported that both fresh bone marrow preparation or adipose-derived stem cell-seeded scaffolds showed superior vascularization and graft function compared to the acellular scaffold in streptozotocin-diabetic rats. Using a novel method to enhance endothelial cell coverage in whole lung scaffolds, Yuan et al. developed a culture protocol with the presence of Rho-associated protein kinase (ROCK) inhibitor (Y27632). They investigated the effect of decellularized whole lung scaffolds on reseeded endothelial phenotypes and functions using single-cell RNA-sequencing analysis. This sequencing analysis demonstrated that primary rodent pulmonary microvascular or human-induced pluripotent stem cell-derived endothelium started to regain the native endothelial phenotypes after transitioning from expansion on culture plates into the decellularized lung scaffold.

Akinnola et al. demonstrated that endothelial cells may exhibit selective tropism during reendothelialization. They reseeded the blood vessels of decellularized mouse lung with rat pulmonary microvascular endothelial progenitor cells (RMEPCs), pulmonary arterial endothelial cells (PAECs), or microvascular endothelial cells (MVECs). They reported that PAECs and MVECs possessed selective tropism for larger vessels or microvasculature, respectively, while RMEPCs lacked site preference and were located to all vascular segments. Additionally, they reported that cells with selective tropism were suboptimal for functional reendothelialization of the vasculature within the whole organ.

Cell sourcing for recellularization has been identified as another challenge for the application of decellularized tissues and organs. Girard et al. obtained stromal vascular fractions by 20 inter-syringe passages and suggested that could be applicable to reseed decellularized tissues. These cells are considered a large source of autologous progenitor cells that can be harvested during liposuction or lipectomies that are classically used in plastic and aesthetic surgery.

Neishabouri et al. highlighted and summarized the different decellularization and sterilization methods, testing decellularization efficiency, and the current challenges and prospects of decellularization.

As another application of decellularized tissues, Narciso et al. used different decellularizing agents to develop a fast and efficient decellularization technique for lung tissue slices attached to a glass slide. This work could be an important tool for cancer research studies on scarce and valuable samples/clinical biopsies as well as to use the decellularized extracellular matrix as a cell culture substrate. Last but not least, Solarte David et al. discussed the natural decellularized ECM, obtained from different tissues, as a promising approach for wound healing. These ECMs could provide a convenient microenvironment through its constituents of cytokines, proteins, and growth factors that could coordinate inflammation, re-epithelialization, and remodelling processes.

This Research Topic presents a snapshot at the cutting edge of tissue and organ decellularization and subsequent recellularization, particularly focusing on the hurdles and unresolved issues that need to be overcome towards the engineering of tissues and organs for clinical transplantation in the near future. The associate editors would like to thank the editors of Frontiers in Bioengineering and Biotechnology for hosting this Research Topic, the reviewers for their valuable help in assessing the contributions, and the authors for contributing their high-quality studies to this Research Topic. The editors hope that their Research Topic will be interesting and useful to the readers and researchers in the fields of tissue engineering and regenerative medicine.
